# Complete Genome Sequences of Lineage III Peste des Petits Ruminants Viruses from the Middle East and East Africa

**DOI:** 10.1128/genomeA.01023-14

**Published:** 2014-10-23

**Authors:** Murali Muniraju, Muhammad Munir, Ashley C. Banyard, Chrisostom Ayebazibwe, Jonas Wensman, Siamak Zohari, Mikael Berg, AravindhBabu R. Parthiban, Mana Mahapatra, Geneviève Libeau, Carrie Batten, Satya Parida

**Affiliations:** a The Pirbright Institute, Pirbright, Woking, Surrey, United Kingdom; b Swedish University of Agricultural Sciences, Uppsala, Sweden; c Animal Health and Veterinary Laboratories Agency, Weybridge, Surrey, United Kingdom; d National Animal Disease Diagnostics and Epidemiology Centre, Entebbe, Uganda; e National Veterinary Institute, Uppsala, Sweden; f CIRAD, UMR CMAEE, F-34398 and INRA, UMR 1309 CMAEE, Montpellier, France

## Abstract

For the first time, complete genome sequences of four lineage III peste des petits ruminants (PPR) viruses (Oman 1983, United Arab Emirates 1986, Ethiopia 1994, and Uganda 2012) originated from the Middle East and East Africa are reported here. The availability of complete genome sequences from all four lineages (I to IV) of the PPR virus (PPRV) would greatly help in a comprehensive understanding of the molecular evolution and emergence of PPRV.

## GENOME ANNOUNCEMENT

Peste des petits ruminants (PPR) is an important infectious viral disease of domestic and wild small ruminants and endemic in the Middle East, Africa, and Asia (1) (http://www.fao.org/docrep/017/aq236e/aq236e.pdf). Phylogenetic analysis of the partial F gene (322 nt) (2), the N gene (255 nt) (3), or the H gene (299 nt) (4) has defined the existence of four distinct lineages (I to IV) of peste des petits ruminants virus (PPRV). Lineages I and II are mainly circulating in west and central Africa, lineage III in the Middle East and East Africa, and lineage IV in Asia and currently in Africa (1). Complete genome sequences of lineages I, II, and IV are available in the literature, whereas only partial genome sequences for F, N, and H genes are available for lineage III (4, 5). Availability of complete genome sequences for all lineages will help in a better understanding of the evolution and spread of PPRV into new geographical regions (6).

The complete genomes of four PPRV isolates from the Middle East, Oman 1983 (7) and United Arab Emirates (UAE) 1986 (8), and from East Africa, Ethiopia 1994 (9) and Uganda 2012, were sequenced in this study following methods described previously (10). The genome size of all four PPRV full genomes reported here are 15,948 nt and the genome organization was the same as that of other PPRV strains. The 3′ ends of the genomes start with a genomic promoter, followed by the transcriptional units of the structural protein genes (N, P, M, F, H, and L) and end with the 5′ antigenomic promoter. Genome and antigenome promoter regions, gene start and stop sequences, and intergenic trinucleotides were present as expected.

Phylogenetic analysis of all the available (at GenBank as of August 2014) complete genome sequences of PPR viruses (EU267273, EU267274, X74443, JX217850, JF939201, FJ905304, NC006383, KC594074, KJ867541, KJ867542, KM212177, and KM091959), including the four viruses sequenced in this study (KJ867540, KJ867543, KJ867544, and KJ867545), has grouped them into four genetically divergent lineages that are similar to the phylogenetic study using partial F, N, and H genes. Complete genomes of PPRV isolates (Oman 1983, UAE 1986, Ethiopia 1994, and Uganda 2012) sequenced in this study were confirmed to be of lineage III. Therefore, this study demonstrated the co-circulation of lineages III and IV in East Africa (5, 11). Further, the availability of complete genome sequences from all four lineages of PPRV will greatly help in a better understanding of molecular evolution and emergence of PPRV.

### Nucleotide sequence accession numbers.

The complete genome of the PPRV isolates have been deposited in GenBank under accession no. KJ867543 (Uganda 2012), KJ867540 (Ethiopia 1994), KJ867545 (UAE 1986), and KJ867544 (Oman 1983).
